# Supplementary material for: Stratifying macrophages based on their infectious burden identifies novel host targets for intervention during Crohn’s disease associated adherent-invasive Escherichia coli infection
Source: Microbiology (Reading). 2024 Jun 25;170(6):001470. doi: 10.1099/mic.0.001470 (PMC11261827; doi:10.1099/mic.0.001470)
Supplement: Uncited Fig. S1. [file mic-170-01470-s001.pdf]

Title

Stratifying macrophages based on their infectious burden identifies novel host targets for intervention during Crohn’s disease associated adherent-invasive *Escherichia coli* infection

Authors

Xiang Li[1], John Cole[1], Diane Vaughan[1], Yinbo Xiao[2], Daniel Walker[3], Daniel M. Wall[1,*]

Supplementary Materials

Supplementary Figures S1-S3

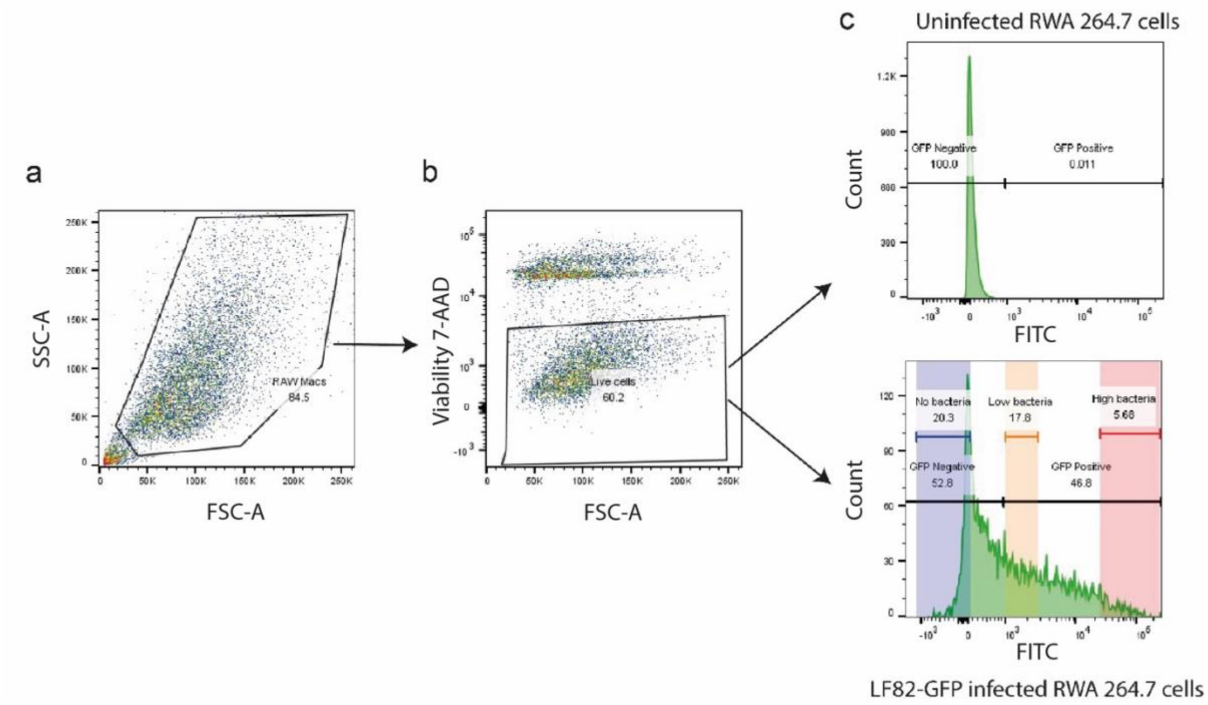

Figure S1: Gating strategy for isolation of LF82::*rpsM*GFP infected RAW 264.7 cells for three different populations (*No*, *Low* and *High*). (**a**) For the isolation of a highly pure RAW 264.7 population, cells were gated on their forward scatter area (FSC-A) and side scatter area (SSC-A), excluding debris from the live gate. (**b**) Dead cells were further excluded based on FSC-A versus the intensity of 7AAD. (**c**) This was followed by gating out three sub-populations of living RAW 264.7 cells according to GFP intensity, resulting in sorting final three populations including cells with no bacterial burden, low bacterial burden and high bacterial burden.

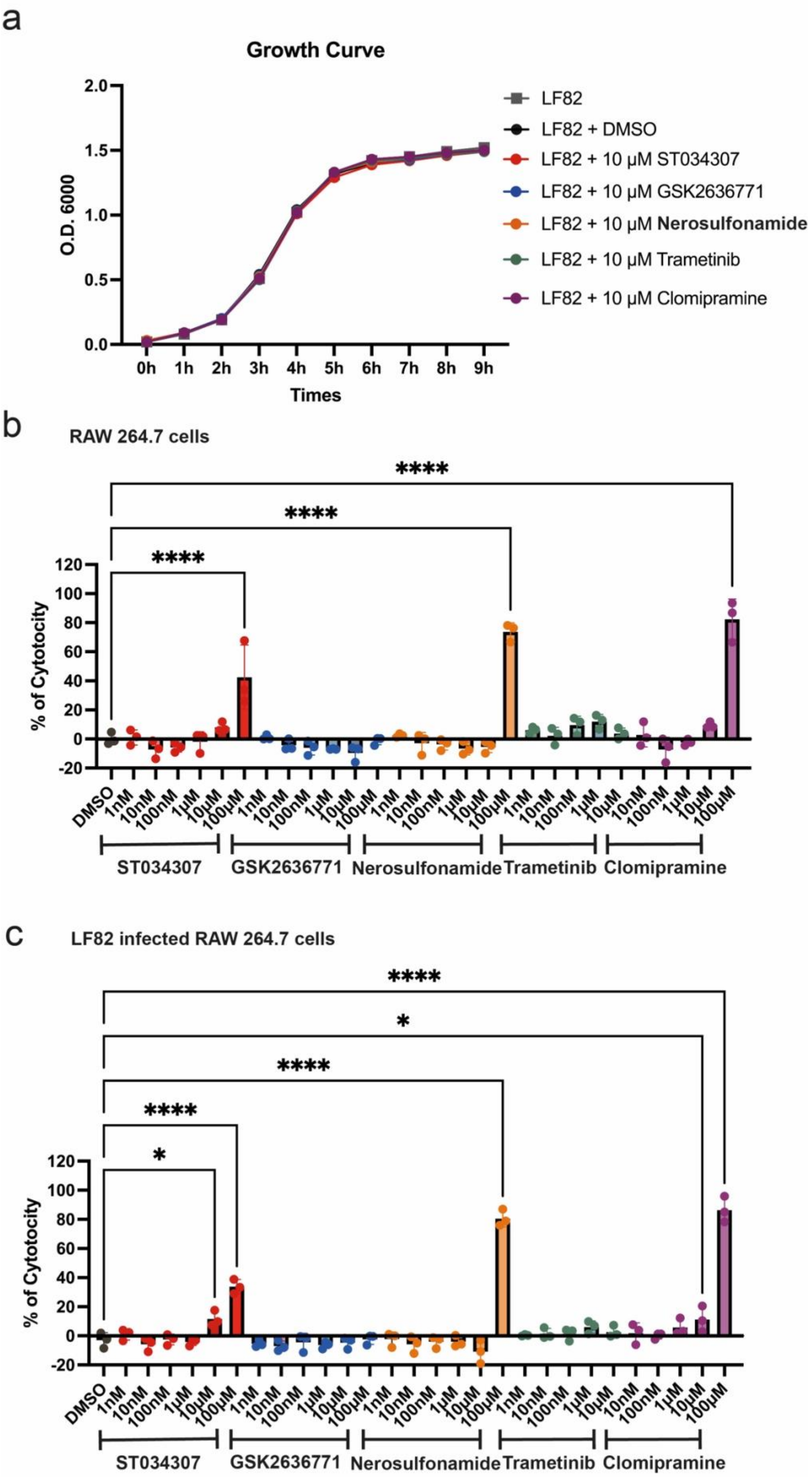
a
Growth Curve
O.D. 6000
Times
0h 1h 2h 3h 4h 5h 6h 7h 8h 9h
0.0 0.5 1.0 1.5 2.0
LF82
LF82 + DMSO
LF82 + 10 μM ST034307
LF82 + 10 μM GSK2636771
LF82 + 10 μM Nerosulfonamide
LF82 + 10 μM Trametinib
LF82 + 10 μM Clomipramine
b
RAW 264.7 cells
****
****
****
% of Cytotocity
-20 0 20 40 60 80 100 120
DMSO 1nM 10nM 100nM 1μM 10μM 100μM
ST034307
GSK2636771
Nerosulfonamide
Trametinib
Clomipramine
c
LF82 infected RAW 264.7 cells
****
*
****
****
*
% of Cytotocity
-20 0 20 40 60 80 100 120
DMSO 1nM 10nM 100nM 1μM 10μM 100μM
ST034307
GSK2636771
Nerosulfonamide
Trametinib
Clomipramine

Figure S2: Effects of the different chemical inhibitors on LF82 growth and cytotoxicity to RAW 264.7 cells. (**a**) Growth curve of LF82 after treatment with DMSO or the five chemical inhibitors (10 μM ST034307, 10 μM GSK2636771, 10 μM Necrosulforamide, 10 μM Trametinib or 10 μM Clomipramine). (**b**) Lactate dehydrogenase (LDH) cytotoxicity assay was undertaken for uninfected or LF82 infected RAW 264.7 cells treated with different concentrations of chemical inhibitors. DMSO, a diluent for the inhibitors, was used as a control. Experimental groups were compared to the control. Statistical significance was determined by one-way ANOVA. *, p <0 0.05. **, $p < 0.01$. ***, $p < 0.0001$.

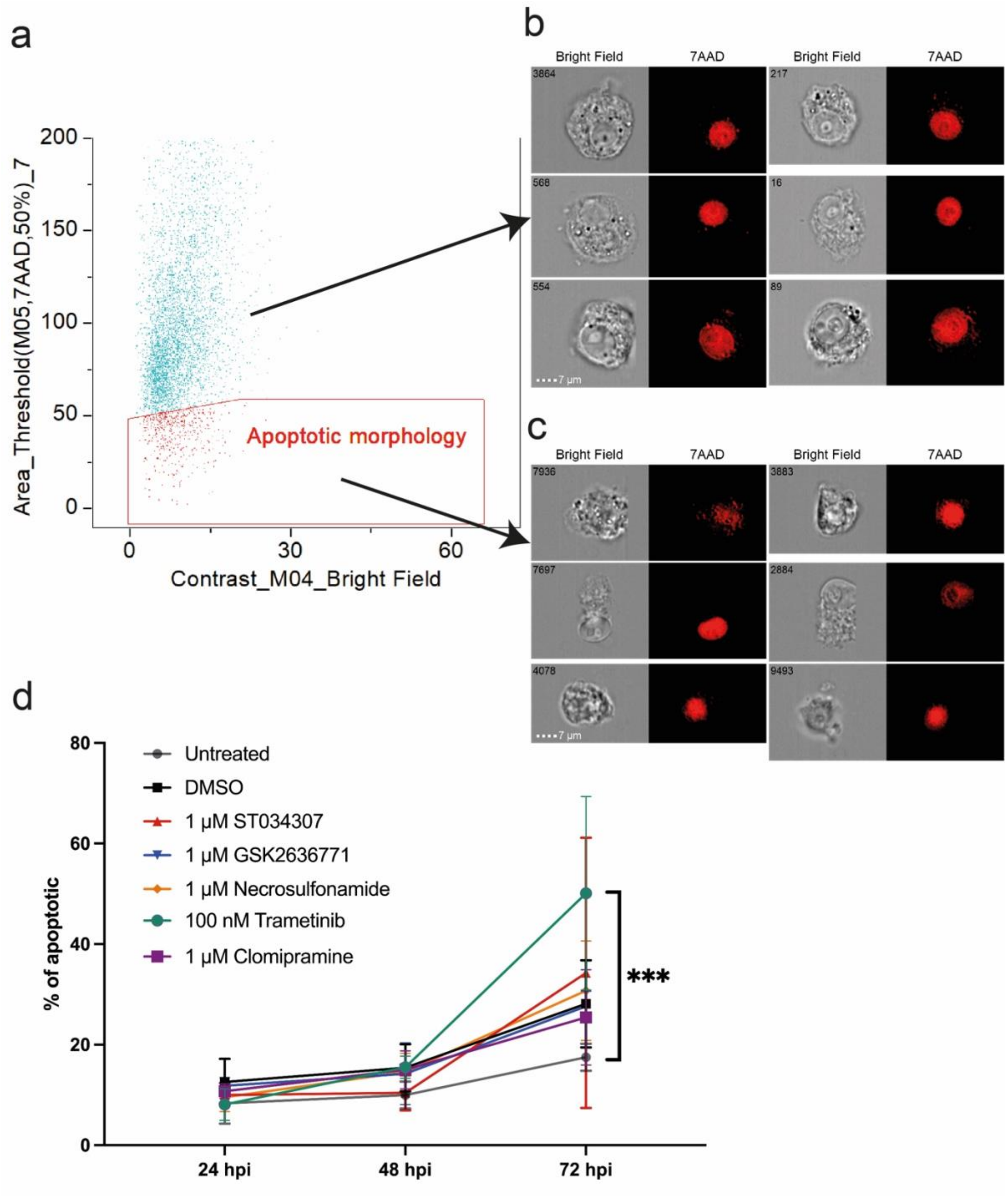

Figure S3: Identification of apoptotic cells using nuclear imagery features. (**a**) Bivariate plot of Area Threshold 50% 7AAD vs. contrast of Brightfield measuring apoptotic morphology based on brightfield and nuclear morphology. Representative imagery of both nonapoptotic cells (**b**) and apoptotic cells (**c**). (**d**) A time course (24, 48 and 72 hpi) of an apoptotic index from GFP-infected RAW 264.7 cells after treatment with DMSO, 1 μM ST034307, 1 μM GSK 2636771, 1 μM Necrosulfonamide, 100 nM Trametinib and 1 μM Clomipramine, respectively. The apoptotic morphology-based indices shown in the graphs are an average of three

biological repeats. Error bar represents the ± SD. Statistical significance was determined by two-way ANOVA. *, p <0 0.05. **, $p < 0.01$. ***, $p < 0.0001$.
